# Crystal Structure of the Neuropilin-1 MAM Domain: Completing the Neuropilin-1 Ectodomain Picture

**DOI:** 10.1016/j.str.2016.08.017

**Published:** 2016-11-01

**Authors:** Tamas Yelland, Snezana Djordjevic

**Affiliations:** 1 The Institute of Structural and Molecular Biology, University College London, Gower Street, London WC1E 6BT, UK

**Keywords:** neuropilin, MAM domain, signaling, X-ray crystallography, calcium binding

## Abstract

Neuropilins (NRPs) are single-pass transmembrane receptors involved in several signaling pathways that regulate key physiological processes such as vascular morphogenesis and axon guidance. The MAM domain of NRP, which has previously been implicated in receptor multimerization, was the only portion of the ectopic domain of the NRPs for which the structure, until now, has been elusive. Using site-directed mutagenesis in the linker region preceding the MAM domain we generated a protein construct amenable to crystallization. Here we present the crystal structure of the MAM domain of human NRP1 at 2.24 Å resolution. The protein exhibits a jellyroll topology, with Ca^2+^ ions bound at the inter-strand space enhancing the thermostability of the domain. We show that the MAM domain of NRP1 is monomeric in solution and insufficient to drive receptor dimerization, which leads us to propose a different role for this domain in the context of NRP membrane assembly and signaling.

## Highlights

• Structural description of the MAM domain of neuropilin-1
• The structure includes a Ca^2+^-binding site stabilizing the jellyroll fold
• The MAM domain of neuropilin-1 is monomeric
• MAM domains engage context-specific surfaces for interaction with other proteins

## Graphical Abstract

**Figure:**
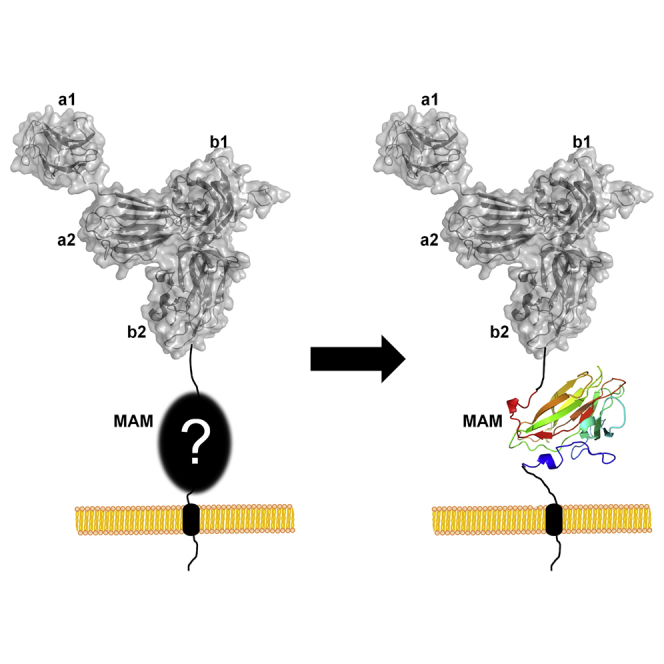

## Teaser

Yelland and Djordjevic complete the structural description of the neuropilin-1 ectopic region by reporting the crystal structure of its MAM domain. The domain adopts a jellyroll fold that is stabilized by a Ca^2+^ ion while forming a molecular surface that is distinct from its structural homologs.

## Introduction

Neuropilins (NRPs) are single-pass transmembrane receptor proteins with a pivotal role in key physiological processes such as vasculogenesis, angiogenesis, axonal guidance, and immunomodulation (Parker et al., 2012a, Djordjevic and Driscoll, 2013). In addition to being essential in development, NRPs have also been implicated in pathologies such as various cancers and proliferative retinopathies (Djordjevic and Driscoll, 2013). Therefore, NRPs have attracted significant scientific interest, because the understanding of their structure, regulation, and molecular basis of signaling mechanisms could be used to inform cancer therapeutics discovery programs targeting these receptors.

NRPs regulate cellular responses to signals mediated by a diverse range of ligands including vascular endothelial growth factors (VEGFs) (Soker et al., 1997, Soker et al., 1998) and semaphorins (Kolodkin et al., 1997). NRPs are able to respond to these diverse ligands owing to the modular organization of their extracellular region (also referred to as ectodomain). Neuropilin-1 (NRP1) and neuropilin-2 (NRP2) exhibit distinct expression patterns; however, they share a common domain organization and 44% amino acid sequence identity in humans (Kolodkin et al., 1997). They are composed of five extracellular structural domains, named **a1**, at the N terminus, then **a2**, **b1**, **b2**, and **c**, followed by a single transmembrane helix and a short cytoplasmic tail. Several research groups investigated the detailed atomic structure of the first four extracellular domains, as well as their interaction with biological or synthetic ligands (Lee et al., 2002, Vander Kooi et al., 2007, Appleton et al., 2007, Jarvis et al., 2010, Parker et al., 2012b, Tsai et al., 2016). It has been shown that domains **a1** and **a2** belong to the structural family of CUB domains and that they are required for interaction with semaphorins (Appleton et al., 2007, Nakamura et al., 1998). NRP domains **b1** and **b2** share structural homology to coagulation factor V/VIII domains and while both are required for maintaining receptor integrity, series of functional and structural studies have demonstrated that VEGFs interact with the NRPs by binding of the C-terminal ends of VEGFs to the specific site on the **b1** domain (Vander Kooi et al., 2007, Tsai et al., 2016).

The membrane proximal **c** domain of NRPs is also called the MAM domain (referred to as such in the rest of the text) based on its sequence homology to the equivalent domains in meprin, A5, and mu-phosphatase (RPTPmu) (Takagi et al., 1991, Beckmann and Bork, 1993). While the structures of the other extracellular domains of NRPs have been investigated extensively, in isolation or in the context of the longer protein constructs, the structure of the MAM domain remained elusive. Currently, there is no evidence that the MAM domain participates directly in an interaction with any of the known ligands for NRPs. Instead, it was proposed that this domain supports dimerization and multimerization of NRP molecules and thus contributes to the formation of signaling receptor complexes (Nakamura et al., 1998). Namely, as the short intracellular domain of NRPs does not display any catalytic activity, NRPs exert their action by combining with other transmembrane receptors, most notably VEGF receptors thereby responding to VEGFs, and plexins, responding to semaphorins. How exactly MAM domains contribute to multimerization and receptor complex formation has not been fully explained and the evidence supporting this view is somewhat weak, relying primarily on the immunoprecipitation experiments carried out on various domain deletion mutants of NRPs (Giger et al., 1998, Nakamura et al., 1998).

We were able to generate a protein variant amenable to crystallization and we now report the first crystal structure of the MAM domain from NRPs, thus completing the structural characterization of the ectodomain of this receptor family. The crystal structure confirms that the domain shares an overall topology with the homologous domain of RPTPmu; however, we see no evidence that the NRP1 MAM domain forms biologically relevant dimers. This observation is also corroborated by the solution studies of the protein. We propose that while the MAM domain on its own is not sufficient to support multimerization of NRP molecules it might contribute to the assembly and regulation of the signaling complexes either by positioning the other extracellular domains of NRPs away from the membrane, and in an orientation suitable for protein/protein interaction, or via glycan-mediated interaction of the MAM domain with other proteins of the extracellular matrix.

## Results and Discussion

### Buffer and Construct Optimization for Crystallization Suggests Calcium Binding

We have previously determined that the specific serine and threonine residues upstream of the predicted MAM domain of NRP1 are glycosylated (Windwarder et al., 2016), producing a non-homogeneous protein sample which was not suitable for crystallization. To overcome this issue all residues that are potential substrates for glycosylation were changed to aspartates to exclude heterogeneity while maintaining negatively charged features of the protein region, thus partially mimicking the effect of glycosylation; this protein variant was named a non-glycosylated MAM (ngMAM) domain. Even though the glycosylation sites were predicted to be located in an unstructured region preceding the residues contributing to the MAM domain fold, prior efforts to remove the glycosylated residues through the generation of truncated constructs resulted in significantly lower expression levels, suggesting that the N-terminal disordered region is necessary for maintaining protein solubility. The ngMAM protein exhibited reduced solubility (25 mg/mL) compared with the wild-type (WT) protein (>150 mg/mL) and this property has proved beneficial for crystallization trials. In all other respects, the ngMAM protein behaved as a WT protein with similar levels of expression and elution profiles on a size-exclusion chromatography column.

**Figure 1:**
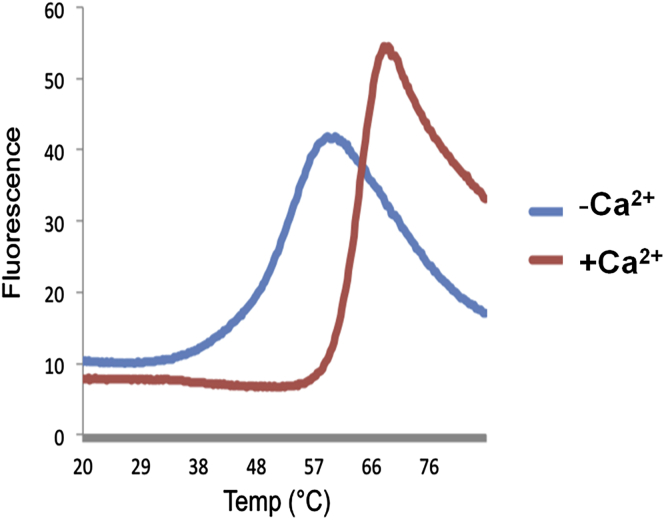
Thermal Stability Assay for Buffer Optimization of the ngMAM Domain The melting temperature of the ngMAM domain (blue curve) increases by ∼10°C on addition of 10 mM CaCl_2_ (red curve).

To further stabilize the protein, buffer optimization was carried out using a thermal melt assay which identified that divalent cations, particularly Ca^2+^ and Mg^2+^, increased protein thermal stability by ∼10°C (Figure 1). Consequently, 10 mM CaCl_2_ was added to the protein solution prior to screening of crystallization conditions.

### Crystal Structure of the MAM Domain of NRP1

**Figure 2:**
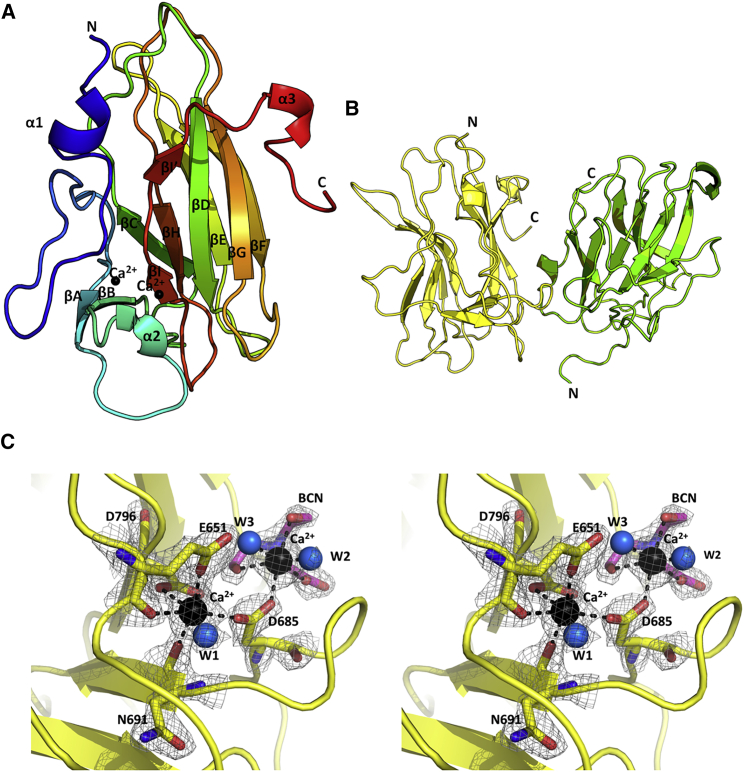
Crystal Structure of the MAM Domain of NRP1 (A) The crystal structure of the MAM domain in cartoon representation colored as a spectrum from blue to red from the N to the C terminus. The structure contains 10 β strands and three short α-helical turns. The two calcium atoms are shown as black spheres. (B) The dimer within the asymmetric unit shown in cartoon representation with one chain in yellow and the second in green. (C) The crystal structure of the MAM domain revealed two calcium-binding sites in close proximity. The calcium-binding sites are shown in stereo view. First Ca^2+^ (black sphere) is coordinated by E651, D685, N691, and D796 as well as a single water molecule (W1). The second Ca^2+^ is coordinated by two water molecules (W2 and W3), D685, and a bicine molecule present within the crystallization condition. The coordinate bonds are shown by black dashed lines. The water molecules are shown as blue spheres while amino acid side chains (yellow) and a bicine molecule (pink) are shown in stick representation. Electron density (2F_o_ − F_c_) is contoured at 1.6 σ and represented as a wire mesh.

The NRP1 ngMAM domain protein crystallized with two molecules in the asymmetric unit (X-ray crystallography statistics are presented in Table 1). The electron density maps allowed model building for residues 639–813 in chain A and 641–815 in chain B while the N-terminal region (residues 628–639), which contains four of the mutated residues that would be glycosylated in the WT protein, as well as the (His)_6_ C-terminal tag, was disordered. This is not surprising given the large number of charged residues in these regions preventing stable secondary structure formation. As the N-terminal region containing mutated glycosylation sites is unstructured and does not contribute to the overall MAM domain protein fold for the rest of the description and discussion we will refer to the MAM domain structure even though the protein used for crystallization was that of a ngMAM variant. The crystal structure of the MAM domain of NRP1 comprises a variable N-terminal region that contains a short α helix connected through an extensive linker to a single β strand (βA), and an adjacent eight-stranded β barrel organized in a jellyroll topology, with the strands named B-I, followed by the short α-helical turn at the C terminus (Figure 2A). The β strands within the domain undergo a significant twist relative to each other such that the βA is almost perpendicular to the βC. The N-terminal α-helical turn may not be a stable structural feature, as it is present in only one of the two monomers within the asymmetric unit. In addition to the polypeptide chain, we were also able to identify two Ca^2+^ ions and a molecule of bicine associated with each of the NRP1 molecules within the asymmetric unit.

The molecular interface observed in the crystal structure (Figure 2B) is unlikely to be biologically significant as determined using the PISA server (http://www.ebi.ac.uk/pdbe/pisa/), which calculated the largest interface between MAM monomers to cover 564 Å^2^ and with a complexation significance score (based on a calculated ΔG upon dimer formation) of 0.0. Therefore the observed dimer in the asymmetric unit is possibly a crystallographic artifact of no physiological relevance. In contrast to the meprin MAM domain (Arolas et al., 2012), which contains five cysteine residues in the MAM domain of NRP1, no intermolecular disulfide bond can be formed as the four cysteine side chains present form intramolecular disulfide bonds, thus eliminating cysteine-dependent oligomerization as a mechanism of NRP1 self-association. However, it has recently been postulated that the integrity of the intramolecular disulfide bonds might regulate NRP oligomerization (Barton et al., 2015). The crystal structure presented here indicates that disruption of the disulfide bonds would affect the stability of the MAM domain and consequently the full ectopic domain.

### Calcium-Binding Site

The initial difference electron density maps contained distinct high-intensity peaks near residues E651, D685, and D796 and these were interpreted as Ca^2+^ ions (Figure 2C). Even though both Mg^2+^ and Ca^2+^ ions were present under the crystallization conditions, the concentration of CaCl_2_ was higher, with an additional 10 mM CaCl_2_ added to the protein buffer used for crystallization. Further confirmation that the ion within the structure was Ca^2+^ came from the analysis of the coordination geometry. A calcium ion is coordinated by a single oxygen from the side-chain carboxyl groups from residues E651 and D685, both oxygen atoms of the side-chain carboxyl group of D796, the backbone carbonyl oxygen atoms from residues E651 and N691 as well as one water molecule (Figure 2C), to give a total of seven coordination interactions with octahedral geometry (as determined using the CMM server, http://www.csgid.org/csgid/metal_sites#home-anchor). This geometry is consistent with a calcium-binding site based on the surveys of deposited crystal structures (Glusker et al., 1996, Harding, 2004) and Fourier transform infrared spectroscopy studies (Nara et al., 2013). In addition, refined distances for metal-ligand oxygen in the structure of NRP1 MAM are around 2.4 Å which is what would be expected for a Ca^2+^/ligand interaction (Zheng et al., 2008), although it could not be excluded that under physiological conditions Mg^2+^ could also bind at the same site. The second Ca^2+^, located within 5.6 Å of the first, is coordinated by four atoms from a bicine molecule present in the crystallization buffer, two water molecules, and an oxygen atom from the β-carboxylate of the D685 side chain, arranged around a Ca^2+^ ion in pentagonal bipyramidal geometry. D685, in fact, bridges the two calcium ions with the β-carboxylate engaged with each of the ions by one of the two oxygen atoms. Given that bicine is a constituent of the crystallization solution, the second Ca^2+^ binding site may not be of a physiological significance. The two Ca^2+^ binding sites are present in both MAM domain molecules within the asymmetric unit and stabilize the domain by bridging the residues from the N-terminal variable region to the first and the last strand of the jellyroll (Figure 2).

**Figure 3:**
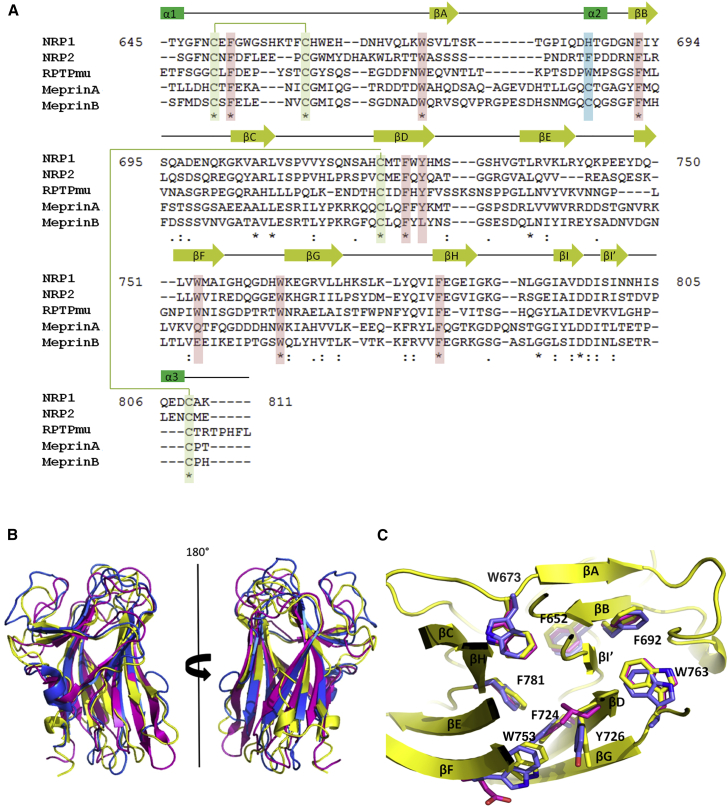
Conservation of Structure of MAM Domains in Relation to Amino Acid Sequence (A) Sequence alignment of the MAM domain from NRPs, RPTPmu, and meprins show the aromatic residues that are highly conserved (red-shaded) between the different proteins. Secondary structure elements present in the NRP1 domain are depicted above the corresponding sequence. Conserved cysteines involved in intramolecular disulfide bonds are highlighted in green shading. Sequence numbering refers to NRP1 MAM domain only. In addition, a solvent-exposed cysteine residue that is present in the meprin protein and involved in disulfide-linked dimerization is highlighted in blue. (B) Overlay of the MAM domains from RPTPmu (blue, residues 1–187; PDB: 2C9A) and meprin (pink, residues 257–428; PDB: 4GWN) to the MAM domain of NRP1 (yellow, PDB: 5L73) in cartoon representation. The secondary structure elements are conserved but there is a large amount of variation within the loop regions. (C) The MAM domain has a conserved hydrophobic core. The MAM domains of NRP1 (yellow), meprin (pink), and RPTPmu (blue) contain a hydrophobic core consisting of aromatic residues F652, W673, F692, F724, Y726, W753, W763, and F781 (NRP1 numbering), shown in stick representation.

The residues contributing to the coordination of the first calcium ion are conserved in the MAM domains from meprin and RPTPmu even though, in both crystal structures, they have been modeled as coordinating to sodium ions. In contrast, it is unlikely that the MAM domain of NRP2 would bind calcium; while the residues corresponding to N691 and D796 are conserved, the other two residues from the binding site, E651 and D685, correspond to an asparagine and a threonine, respectively (Figure 3A). Previously, it was reported that the NRP1 **a1a2** domains also contain a calcium-binding site (Appleton et al., 2007) and it has recently been shown that the **b1** domain of NRP2 has a specific Zn^2+^ ion binding site (Tsai et al., 2016). Therefore, the signaling potential of NRPs might be additionally regulated by homeostasis of divalent ions.

### Comparison with the MAM Domains from RPTPmu and Meprin

Overlaying the MAM domain structure from RPTPmu (PDB: 2C9A, residues 1–187, 25% sequence identity to NRP1) (Aricescu et al., 2006) and meprin (PDB: 4GWM, residues 257–428, 29% sequence identity to NRP1) (Arolas et al., 2012) to that of NRP1 gives an RMSD for Cα atoms of 1.6 Å and 1.7 Å, respectively (Figure 3B). The β strands overlap very well while the flexible loops are the main source of variation between the three structures. It appears that the central β barrel of the MAM domains provides a scaffold that displays the loop regions which are crucial determinants of the function of the individual domains in the context of the various full-length proteins (Aricescu et al., 2006).

This conclusion is reinforced by the analysis of the MAM domain structures which revealed a highly hydrophobic core spanning the entire length of the fold. In NRP1 the aromatic residues F652, W673, F692, F724, Y726, W753, W763, and F781 are arranged in a coordinated manner to optimize π-stacking interactions between the aromatic side chains (Figure 3C). In the core of RPTPmu these residues are conserved and superimpose very well with the NRP1 residues. In meprins, most of the corresponding residues are also conserved with the exception of the NRP1 W753 which, in meprins, is located at the solvent-exposed edge of the domain and corresponds to Q/E (for meprinA/meprinB) (Figure 3A).

Further analysis of sequence conservation between the three proteins reveals that, apart from the residues contributing to the hydrophobic core there is only one sequence stretch of significant homology, spanning NRP1 residues G791 to I800. Interestingly, this area includes the key residue (D796) contributing to the binding of the Ca^2+^ ion. Despite the conserved central β structure, connecting loops are very different in both amino acid composition and the length. Even when the surface loops of equivalent lengths are considered, such as the loop connecting βE to βF, the 3D positions of the equivalent Cα residues are up to 7 Å apart, thus creating a distinct molecular surface for each of the proteins.

### MAM Domains Utilize Different Molecular Surfaces to Engage in Protein/Protein Interactions

**Figure 4:**
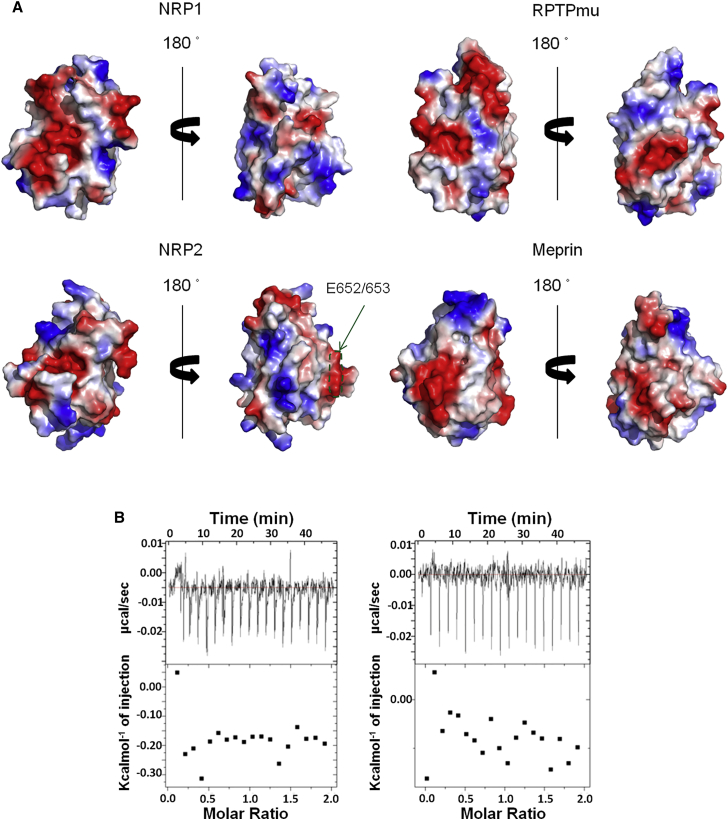
Different MAM Domains Might Be Engaged in a Distinct Set of Protein/Protein Interactions (A) Molecular surface electrostatic potential of the MAM domains from NRP1, NRP2, RPTPmu, and meprin. The structures are shown in the same orientation. NRP2 MAM was modeled based on the structure of NRP1 reported here. For the surface of NRP2, the green arrow points to the region, recently described as being engaged in interaction with polysialyltransferase (Bhide et al., 2016). See also Figure S1. (B) NRP1 MAM exhibits a monomeric structure in solution, as suggested by isothermal titration calorimetry experiments. Titration of NRP1 MAM domain into a buffer solution at 15°C (left) and 20°C (right). Top panels show raw data while the bottom panels contain a binding isotherm obtained by integrating the peaks from the top panel.

Differences in the structures of the loop regions give rise to the significantly different molecular surfaces of the various MAM domains (Figure 4A). Comparison of the electrostatic surface potentials for the structures of NRP1, RPTPmu, and meprin MAM domains demonstrates that while all structures display a mixture of polar and hydrophobic patches there are clear differences in the molecular shapes and the distribution of charges within the three electrostatic surface potentials, suggesting that the three homologous domains would be engaged in a distinct set of intra- or intermolecular protein/protein interactions.

MAM domains have been widely implicated in protein oligomerization and receptors assembly; however, the mechanisms they employ to facilitate protein/protein interactions remain under-determined. The structures of the RPTPmu MAM domain in a context of a MAM-Ig domain (PDB: 2C9A) (Aricescu et al., 2006), and as a part of the full-length ectodomain (PDB: 2V5Y) (Aricescu et al., 2007), indicated that the N-terminal MAM domain is involved in receptor dimerization but not through homo-domain interactions; instead, the domain binds to an FNIII domain of a neighboring molecule in a pH-dependent manner (Aricescu et al., 2006). Furthermore, within the antiparallel dimer of the RPTPmu ectodomain, both the MAM and Ig domain contribute to the molecular surface interacting with the FNIII domain (Aricescu et al., 2007). In NRPs, the MAM domain is located proximal to the membrane and thus, despite the similarity to the fold of the RPTPmu MAM domain it would be unlikely that similar set of domain/domain interactions would also be observed within the context of a full-length NRP1.

Even though it had been postulated that the MAM domain of NRP1 would be engaged in homo- and hetero-dimerization, the evidence for this hypothesis is weak, relying mostly on cell-based experiments with NRP1 variants where individual domains have been deleted (Nakamura et al., 1998). Using size-exclusion chromatography, we have previously estimated that an ∼40 μM WT glycosylated MAM domain is monomeric in solution (Windwarder et al., 2016), while the structure presented here contains two molecules in the asymmetric unit (Figure 2B). To further assess the potential of the NRP1 MAM domain to form homo-dimers or homo-oligomers we used isothermal titration calorimetry for which a highly concentrated purified MAM domain (900 μM) was titrated into a buffer solution (Figure 4B). If the MAM domain was present as a dimer at the initial concentrations, injection of the concentrated MAM domain to the buffer chamber would result in a 200-fold dilution of the initial concentration down to <5 μM, and if the protein existed primarily as a dimer at the initial concentration, the protein molecules would dissociate at the lower concentration with an accompanying enthalpy change. However, no measurable heats of dissociation were detected by the instrument. This result suggests that if the MAM domain was to homo-oligomerize then the self-association affinity would be weak and with a corresponding dissociation constant higher than 900 μM. Our experiments, in agreement with the previously reported light-scattering data (Appleton et al., 2007), do not demonstrate self-association of the MAM domain.

We, therefore, propose a different role for the MAM domain in the context of NRP signaling, where it would act as an insulator, shielding the rest of the ectodomain from the membrane environment and positioning other domains (**a1a2b1b2**) for their interaction with partner co-receptors. Alternatively, it may serve to modulate signaling and multimerization events through interactions with other binding partners, such as galectins (GALs). It has been shown that GAL-1 (Camby et al., 2006) affects both angiogenesis and neuronal development (Quinta et al., 2014) and that it binds to NRP1. Although the molecular basis of this interaction is unknown, it is possible that GAL-1 associates with the glycans upstream of the NRP1 MAM, thereby bridging the NRP ectodomains. In a recent report (Bhide et al., 2016), two glutamate residues in the NRP2 MAM domain were identified as the recognition site for the polysialyltransferase (an enzyme that specifically modifies NRP2 but not NRP1). In our molecular model of the NRP2 MAM domain these residues are located on the unique protrusion of the molecular surface (Figure 4A), further supporting our suggestion that, in NRPs, the MAM domains interact with the specific regulatory proteins by utilizing distinct molecular surfaces.

## Experimental Procedures

### Expression and Purification of the MAM Domains

A gene construct coding for amino acid residues 628–813 of human NRP1 was cloned into the vector pOPINTGneo (Berrow et al., 2009). The DNA was cloned in frame with the secretion leader sequence to ensure that the resulting protein was exported through the secretory pathway. The corresponding protein product referred to as a “MAM domain,” was used for analytical size-exclusion chromatography and isothermal titration calorimetry experiments.

For the production of a ngMAM domain, the gene construct, cloned into the vector pSecTag2 A (Thermo Fisher Scientific), was purchased from Life Technologies. In this expression construct, the codons corresponding to residues T629, T633, S637, T638, S641, and T645 were replaced with the codons for aspartate. The mutated residues removed the O-linked glycosylation sites to produce a homogeneous protein sample suitable for crystallization. The protein was cloned in frame with the Ig K-chain leader so that the MAM domain was exported through the native secretory pathway. A non-cleavable (His)_6_-purification tag was fused to the C terminus.

All large-scale expression protocols were carried out as follows: transfection of the 1 L cultures of FreeStyle HEK293-F cells (Thermo Fisher Scientific) at a density of 1.0–1.4 × 10^6^ cells/mL was performed using 1.25 mg of DNA and 1.87 mg of polyethylenimine (Sigma) in 40 mL of OptiPro (Life Technologies) supplemented with 4 mM L-glutamine (Sigma). Cells were incubated at 37°C, shook at 125 rpm for 4 days before being harvested by centrifugation at 1,000 × *g* for 10 min. The supernatant was filtered and loaded onto a 1 mL Ni^2+^-ion affinity HisTrap column (GE Healthcare). The column was washed with 10 column volumes of a buffer containing 20 mM Tris (pH 8.0) and 100 mM NaCl before the elution step with 5 column volumes of the buffer containing an additional 300 mM imidazole. The protein was further purified by size-exclusion chromatography on a Superdex S200 10/300 GL column (GE Healthcare). All protein concentrations were estimated based on absorbance at 280 nm.

### Crystallographic Studies

#### Crystallization

Purified ngMAM protein was concentrated to 15 mg/mL using centrifugal concentrators (10 kDa molecular-mass cutoff, Vivaspin, Vivascience, Thermo Fisher Scientific) before adding CaCl_2_ to a final concentration of 10 mM. Crystallization screening was performed using a mosquito (TTP Labtech) and a sparse matrix kit from Hampton Research (Index), Molecular Dimensions (Morpheus, PACT and JCSG), and QIAGEN (PEGS II). Rod-shaped crystals (∼100 μM in length) grew at 16°C, in a solution containing 0.06 M MgCl_2,_ 0.06 M CaCl_2_, 0.1 M Tris (base), 0.1 M bicine (pH 8.0), 12.5% MPD, 12.5% PEG 1000 and 12.5% (w/v) PEG 3350. The crystals were flash frozen directly in liquid nitrogen prior to data collection.

#### Data Collection and Structure Determination

Diffraction data were collected at Diamond Light Source, beamline I04. Data were processed using Xia2 (Winter, 2010) and reindexed with Pointless (Evans, 2011) before phasing with the molecular replacement method using Phaser (McCoy et al., 2007). For a search model, the structure of the MAM domain from RPTPmu (PDB: 2C9A, residues 21–177) was used (omitting all water molecules and glycans). We were unable to obtain the solution until the following loop and flexible regions were omitted: residues 21–36, 51–67, 89–95, 106–108, 121–125, 147–154; the side chains of the remaining residues were cut to Cβ using Chainsaw (Stein, 2008). The structure was built using iterative rounds of model building in Coot (Emsley et al., 2010) and a restrained refinement routine in Refmac5 (Murshudov et al., 1997). All figures of crystal structures and molecular surface calculations were prepared using PyMol (http://www.pymol.org).

### Isothermal Titration Calorimetry

MAM domain dissociation experiments were performed using a MicroCal iTC200 (Malvern) instrument. The sample cell was filled with buffer (20 mM Tris [pH 8.0], 100 mM NaCl) and the syringe contained 900 μM of the MAM domain in an identical buffer solution. The MAM domain solution was then titrated into the buffer with one 0.4 μL injection that was followed by 19 injections of 2 μL. The experiments were performed at 15°C and 20°C with a stirring rate of 1,000 rpm.

### Thermostability Assay

Each well in a 96-well plate contained SYPRO orange (Sigma-Aldrich) at a final 1× concentration, 5 μM of the ngMAM domain, a 2 μL solution from a single condition from the Hampton Research crystallographic additive screen and a buffer (10 mM Tris [pH 8.0], 100 mM NaCl) up to a total volume of 20 μL. The plate was placed in a LightCycler 480 II (Roche) and the samples were heated from 10°C to 90°C at a rate of 5°C per min. Fluorescence was monitored at 570 nm.

### Molecular Modeling of the NRP2 MAM Domain

Generation of the NRP2 MAM domain model structure was performed using MODELLER v9.16 (Webb and Sali, 2014) and using the NRP1 MAM domain as a template (35% sequence identity). Sequence alignments and model generation was performed following the online manual methodology (https://salilab.org/modeller/tutorial/).

## Author Contributions

T.Y. and S.D. designed the experimental approaches. T.Y. carried out the experiments. S.D. supervised the work and assisted with data analysis. T.Y. and S.D. wrote the manuscript.

## Figures and Tables

**Table 1:** Data Collection and Refinement Statistics

**Data Statistics**	

Space group	P 2_1_ 2_1_ 2_1_
Cell dimensions	
a, b, c (Å)	46.17, 59.56, 136.65
α, β, γ (°)	90.00, 90.00, 90.00
Resolution (Å)	46.17–2.24 (2.31–2.24)
R_merge_	0.11 (0.41)
Completeness (%)	98.3 (96.5)
Multiplicity	6.2 (6.0)
CC_1/2_	0.997 (0.909)
I/σ (I)	16 (5.4)

**Refinement**	

Total no. of observations	114,824
Total no. unique	18,498
R_work_/_free_	17.47/22.82
RMSD	
Bonds (Å)	0.011
Angles (°)	1.553
Mean B factors (Å^2^)	25.33
Ramachandran plot (%)	
Residues in favored region	95.13
Residues in allowed region	3.72
Residues in disallowed region	1.15

Numbers in parentheses refer to the highest-resolution data shell. RMSD, root-mean-square deviation.

## Accession Numbers

The coordinates have been deposited with the PDB under accession code PDB: 5L73.

## Supplemental Information

Document S1. Figure S1

Document S2. Article plus Supplemental Information
