# Supplementary material for: Crystal Structure of the Neuropilin-1 MAM Domain: Completing the Neuropilin-1 Ectodomain Picture
Source: Structure. 2016 Nov 1;24(11):2008–15. doi: 10.1016/j.str.2016.08.017 (PMC5104691; doi:10.1016/j.str.2016.08.017)
Supplement: Document S1. Figure S1 [file mmc1.pdf]

**Structure, Volume *24***

## Supplemental Information

# Crystal Structure of the Neuropilin-1 MAM Domain: Completing the Neuropilin-1 Ectodomain Picture

**Tamas Yelland and Snezana Djordjevic**

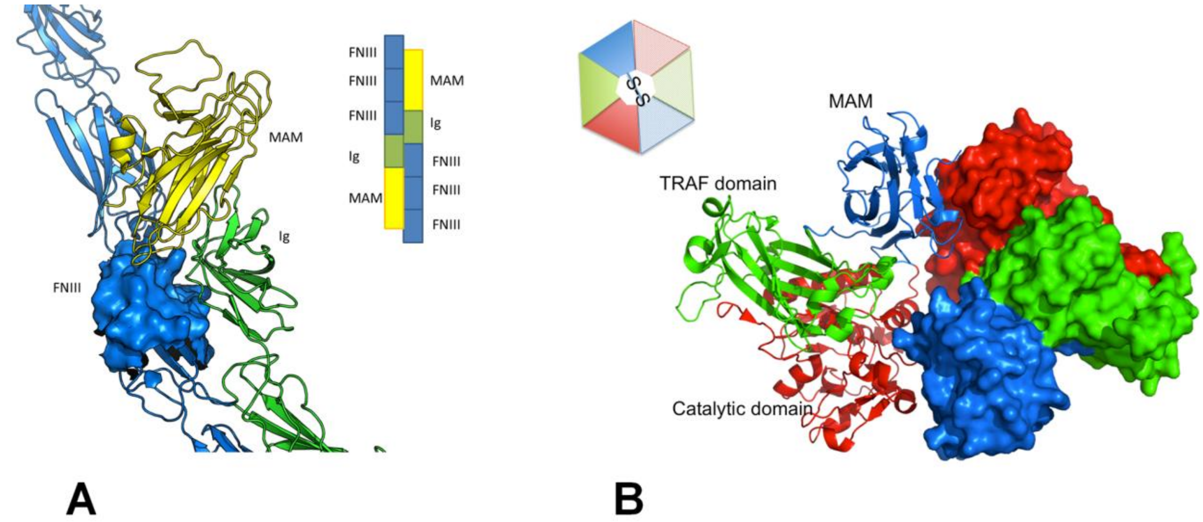


**Figure S1, related to Figure 4. MAM domain interactions of the MAM domain of RPTPmu and meprin** (**A**) In RPTPmu (PDB: 2V5Y) the MAM and neighbouring Ig domain form an interface which recognises the FNIII domain from a separate RPTPmu molecule. (**B**) A meprin dimer (PDB: 4GWM) where one monomer is shown in cartoon representation and the second as in surface representation. The catalytic domain is shown in red, the MAM domain in blue and the TRAF domain in green. The MAM domain from meprin forms an inter-chain disulphide bond with another MAM domain as well as non-covalent interactions with a neighbouring catalytic domain from another meprin molecule.
